# Rare Abdominopelvic Complications in a Hysterectomized Donor with Retained Fallopian Tubes: A Cadaveric Case Study

**DOI:** 10.51894/001c.144475

**Published:** 2025-10-01

**Authors:** Lauren Jernstadt

**Affiliations:** 1 College of Osteopathic Medicine Michigan State University

## 87

### INTRODUCTION

Post-operative adhesions are a common complication of abdominopelvic surgical procedures, including a hysterectomy. However, little is known about post-surgical tissue growths atypical of fibrous adhesions. This case study evaluates a hysterectomized cadaveric specimen that revealed intra-abdominal adhesions and bilateral abnormal tissue masses upon dissection and seeks to evaluate the causes of these abnormalities and their potential consequences.

### CASE DESCRIPTION

During a routine dissection of an 80-year-old female cadaveric donor, evidence suggesting a previous hysterectomy was discovered with bilateral retained fallopian tubes and no gross evidence of retained ovaries bilaterally. A comprehensive abdominopelvic cavity examination revealed round ligaments were attached to the superolateral poles bilaterally of the vaginal vault. Bilateral, relatively symmetrical masses were found originating from the anterior pelvic wall, with the left mass (5.1 cm in length) attached to the left round ligament and the right mass (10.7 cm in length) associated with a segment of the sigmoid colon and rectum. Histologic analysis of the bilateral masses is being conducted to determine potential etiology. Additional pathological findings included a proximally elongated rectum measuring 21.5 cm, a visible adhesion attaching a portion of sigmoid colon to the pelvic cavity wall, a section of sigmoid megacolon, and bilateral, smooth adipose herniations through the left and right superficial inguinal rings measuring 3.6 cm (left hernia) and 4.7 cm (right hernia) in length. Primary cause of death was listed as cerebral infarction with flaccid hemiplegia affecting the right dominant side. 

### DISCUSSION/CONCLUSIONS

While intra-abdominal adhesions represent a major cause of post-operative complications following a hysterectomy, more research is required on etiologies of other rare anatomic complications such as those aforementioned in this study. The results of this case study contribute to the understanding of abnormal post-hysterectomy complications and further elucidate methods of detection, management, and prevention of these complications. Our case study aims to achieve this contribution through gross examination and histopathological analysis to better understand their etiology. By sharing these findings, the authors hope to encourage collaboration among healthcare professionals, enhancing the understanding of such medical presentations and optimize management of patients with similar anatomical variations.

